# Adaptive Sparse Detector for Suppressing Powerline Component in EEG Measurements

**DOI:** 10.3389/fpubh.2021.669190

**Published:** 2021-05-07

**Authors:** Bin-qiang Chen, Bai-xun Zheng, Chu-qiao Wang, Wei-fang Sun

**Affiliations:** ^1^School of Aerospace Engineering, Xiamen University, Xiamen, China; ^2^College of Mechanical and Electrical Engineering, Wenzhou University, Wenzhou, China

**Keywords:** EEG, spare representation, fourier transform, powerline interference, basis pursuit

## Abstract

Powerline interference (PLI) is a major source of interference in the acquisition of electroencephalogram (EEG) signal. Digital notch filters (DNFs) have been widely used to remove the PLI such that actual features, which are weak in energy and strongly connected to brain states, can be extracted explicitly. However, DNFs are mathematically implemented via discrete Fourier analysis, the problem of overlapping between spectral counterparts of PLI and those of EEG features is inevitable. In spite of their effectiveness, DNFs usually cause distortions on the extracted EEG features, which may lead to incorrect diagnostic results. To address this problem, we investigate an adaptive sparse detector for reducing PLI. This novel approach is proposed based on sparse representation inspired by self-adaptive machine learning. In the coding phase, an overcomplete dictionary, which consists of redundant harmonic waves with equally spaced frequencies, is employed to represent the corrupted EEG signal. A strategy based on the split augmented Lagrangian shrinkage algorithm is employed to optimize the associated representation coefficients. It is verified that spectral components related to PLI are compressed into a narrow area in the frequency domain, thus reducing overlapping with features of interest. In the decoding phase, eliminating of coefficients within the narrow band area can remove the PLI from the reconstructed signal. The sparsity of the signal in the dictionary domain is determined by the redundancy factor. A selection criteria of the redundancy factor is suggested via numerical simulations. Experiments have shown the proposed approach can ensure less distortions on actual EEG features.

## Introduction

Electroencephalography (EEG) aims at measuring potentials that reflect the electrical activity of the human brain (1). It has been recognized as a powerful tool in psychophysiology due to its high temporal resolution and sensitivity to index different functional brain states (2). However, because of imperfect measurement conditions, noises are likely to be incorporated in the records of EEG. For instance, EEG signals in actual measurements can often be exposed to strong powerline interferences (PLIs) at 50 or 60 Hz, which is originated from AC power (3). In laboratory environments, good shielding measures, such as shielded rooms, can be helpful to reduce the influence of PLI. But shielding measures are usually impractical for healthcare practices of EEG monitoring via mobile instrument such as wearable devices (4, 5).

In practical applications, solutions to remove the PLI can be roughly categorized into two types, the hardware ways and the software ways (6). The hardware ways are referred to active electrodes with integrated analog filters, but a significant residual interference still remains. In contrast, the software based solutions are more flexible and they can be combined with hardware based techniques. To achieve a more robust performance of the PLI removal, novel approaches based on digital signal processing techniques are more popular and have been extensively studied (7–9). The digital notch filter (DNF) at the powerline frequency, which is designed based on the theory of traditional Fourier analysis, has been widely employed to reduce PLI in biomedical measurements (10). In spite of its effectiveness, side effects of the DNF are also reported in related researches. Firstly, the stop band of the DNF is difficult to be selected. Although the main frequency of PLI stays at 50 or 60 Hz nominally, there is always a variation of ±2Hz in the actual power system. Secondly, the sampling parameters also affect the filtering results. A phenomenon of energy leakage is likely to be produced by sinusoidal waves whose sampling does not satisfy the full period sampling condition (11). Thirdly, the actual EEG features cover a comparatively broad range in the frequency domain. Thus, overlapping between the spectral counterparts of PLI and those of the EEG features is inevitable. As a result, DNF is not a perfect tool for removing PLI.

Multiresolution analysis (MRA) is a powerful tool to characterize non-stationary and transient components. The past three decades have witnessed the rapid development of MRA. As a concrete example of MRA, discrete wavelet transform (DWT) is implemented using a scaling function and a wavelet function (12). DWT has been widely utilized to separate signal components in many scientific researches. On the other hand, flexible DWTs, based on parameter optimization schemes, are also employed to extract transient features (13). In the field of biomedical signal processing, wavelet transform can reveal weak features related to the transient nature of biomedical measurements which are not so obvious by using spectrum analysis. In the literature, it has been reported that DWT can be used as an effective tool in dealing noises such as PLI and baseline wandering from a corrupted biomedical measurement (14). DWT can be also combined with artificial intelligence methods (15, 16), such as deep learning and support vector machine, to realize signal classification applications. However, in suppressing the PLI components, which are more similar to stationary contents in waveform, DWT may be not perfect. Recently, compressed sensing and sparse representation, which still rely on the idea of signal representation, emerge as enhancements to the conventional DWT.

In this paper, to achieve a better PLI suppressing performance, we proposed a novel adaptive detector (ASD). It can be regarded as an improvement to fast Fourier transform, which is a classical spectral analysis tool. Within the proposed method, a redundant Fourier dictionary, developed from the orthogonal Fourier basis, is employed as an over-complete dictionary. The linear combination coefficients with respect to the redundant dictionary are optimized to achieve a sparse representation. Therefore, a sinusoidal wave can be expressed as a combination of a very limited number of sinusoidal atoms. Due to the narrow band property of PLI in the spare representation, they can be more easily isolated from other contents. The performance of the proposed method is verified by numerical simulations and a case study of actual EEGs.

## Materials and Methods

### Signal Modeling and Notations

For the convenience of discussion, we use the following notations for mathematical argument. Let {*x*(*n*)|, *n* ∈ ℤ^+^⋃ {0}} be a corrupted EEG signal containing both the actual brain potential waveform *s*(*n*) and the PLI component *p*(*n*). That is to say, *x*(*n*) = *s*(*n*) + *p*(*n*). Let the sampling length and the sampling frequency of *x*(*n*) be denoted as *N* and *f*_*s*_. The estimated signal after PLI cancellation using some specific algorithm is expressed as $\tilde{s}\left(n\right)$. In this article, *p*(*n*) is modeled as a simple harmonic wave characterized by the harmonic parameters of amplitude (*Amp*_*pli*_), frequency (*f*_*pli*_), and initial phase (φ_*pli*_).

(1) $$\begin{array}{l} p(n)=Amp_{pli}\cdot \cos(2\pi f_{pli}(n-1)\Delta t)+\varphi_{pli}) \end{array}$$

where the time interval Δ*t* = 1/*f*_*pli*_. To remove PLI, an important task lies in accurate estimation of these harmonic parameters such that a compensation signal can be reconstructed. The actual EEG waveform *s*(*n*) is by nature non-stationary and its spectrum covers a comparatively wide range of area in the spectral domain, which is essentially different from that of *p*(*n*).

In optimization theory, the norms of *x*(*n*) are indispensable. Two types of commonly used norms are the ℓ_1_ and ℓ_2_ norms. They are computed using the following formulae.

(2) $$\begin{array}{l} \ell_{1}\;norm:\;||x{\|}_{1}:=\overset{N-1}{\underset{i=0}{\sum }}|x\left(i\right)|\\ \ell_{2}\;norm:\;\|x{\|}_{2}:={\left(\overset{N-1}{\underset{i=0}{\sum }}{\left|x\left(i\right)\right|}^{2}\right)}^{1/2} \end{array}$$

The ℓ_1_ is essential to ensure sparse representation using redundant dictionaries. The ℓ_2_ is strongly connected to least squares approximation, and it is also known as the Euclidean distance.

### Notch Filters Based on Fourier Transform Theory

Fast Fourier transform (FFT) is a fundamental tool for discrete signal analysis. It is an orthogonal decomposition of input signal *x*(*n*) of length *N* via a orthonormal basis Φ_*Fourier*_.

(3) $$\begin{array}{l} \Phi_{{\;}_{Fourier}}^{\;}=\frac{1}{\sqrt{N}}\left[\phi_{0}\;\phi_{1}\;\ldots \phi_{N-1}\right] \end{array}$$

The *k*-th sinusoidal atom in the Fourier dictionary can be defined as

(4) $$\begin{array}{l} \phi_{k}\;=\;{\left(e^{\text{j}\cdot \frac{2\pi }{N}\cdot k\cdot 0},e^{\text{j}\cdot \frac{2\pi }{N}\cdot k\cdot 1},e^{\text{j}\cdot \frac{2\pi }{N}\cdot k\cdot 2},\ldots ,e^{\text{j}\cdot \frac{2\pi }{N}\cdot k\cdot \left(N-1\right)}\right)}^{\text{T}} \end{array}$$

where $\text{j}=\sqrt{-1}$ is defined as the imaginary number and *T* means the transpose operation in matrix algebra. That is to say, the frequency of the sinusoidal atom ϕ_*k*_ is 2π(*k* − 1)/*N*. A uniform step of 2π/*N* is assumed for angular frequencies of two adjacent atoms in the Fourier dictionary. The transform coefficient is computed using the following formula

(5) $$\begin{array}{l} c_{k}\;=\;\phi_{k}^{H}\;x, \end{array}$$

where *H* means conjugate transpose in complex analysis. The representation coefficients of a input signal *x* with respect to the dictionary Φ_*Fourier*_ is expressed using the matrix operation of $\Phi_{Fourier}^{H}\;x$. By using the above transformation, the input signal is expressed as the sum of a few sinusoidal waves with different frequencies, which is expressed as

(6) $$\begin{array}{l} x\;=\;\overset{N-1}{\underset{k=0}{\sum }}c_{k}\phi_{k}. \end{array}$$

The above Equation is also named as the inverse fast Fourier transform (IFFT). For a sinusoidal wave whose sampling parameters satisfy the full period sampling condition, it is mapped to a single spectral line (11). Otherwise the phenomenon of energy leakage occurs and causes dense representation of the sinusoidal wave in the frequency domain.

The method of digital notch filter is theoretically based on the Fourier transform theory. Either finite impulse response (FIR) filters or infinite impulse response (IIR) filters can be designed for conducting PLI removal applications. Some mature development toolkit for designing notch filters can be found in commercial software of numerical computation. By specifying a few parameters, notch filters can be designed conveniently. Despite the effectiveness reported in the literature, these filters also suffer a few drawbacks and may cause distortions on the extracted EEG features. Owing to frequency variations existed in the PLI, it is not possible to design a uniform DNF with pre-determined parameters. Hence, during the implementation of DNFs, strategies allowing self-adaptivity of parameters should be considered.

### Redundant Fourier Dictionary for Spectral Analysis

In the FFT spectrum, the spectral lines are sampled at the frequency of *k*·*f*_*s*_/*N*, in which *k* = 0, 1, …, *N*−1. The interval between adjacent spectral lines are uniform. Just as the digital signal can be regarded as a sampling of the analog signal, the FFT spectrum can be also interpreted as a sampling of the continuous Fourier spectrum. For an arbitrary frequency of *f*_*a*_, the associated Fourier coefficient can be calculated using the following formula

(7) $$\begin{array}{l} X\left(f_{a}\right)=\overset{N-1}{\underset{k=0}{\sum }}x\left(k\right)e^{-\text{j}\cdot 2\pi f_{a}k\Delta t}, \end{array}$$

in which Δ*t* = 1/*f*_*s*_. Besides the FFT sampling grids mentioned above, additional Fourier coefficients can be computed in order to allow insightful investigations. Because redundancy is introduced in signal representation, the relevant dictionary is called as redundant Fourier Dictionary (RFD). A few efficient implementation using RFD have been developed, such as Goertzel algorithm (17), chirp-Z transform (18) and zero padded FFT (19). Among these implementations, the combination of zero padding and FFT algorithm is usually adopted for computing redundant Fourier spectra, in which the RFD is composed of uniformly spaced spectral bins. Although redundancy is beneficial in revealing information in the frequency domain, the corresponding representations are likely to be dense. Therefore, post-processing steps are usually required to estimate harmonic parameters of PLI.

In this paper, in order to sparsely represent a signal, a tight dictionary composed of redundant Fourier atoms is employed. For a *N*-point digital signal, a tight Fourier dictionary *A* containing *K* atoms (*K* > *N*) is defined as

(8) $$\begin{array}{l} A=\left(\begin{array}{cccc} {\tilde{\phi }}_{0} & {\tilde{\phi }}_{1} & \ldots & {\tilde{\phi }}_{K-1} \end{array}\right), \end{array}$$

in which the angular frequency associated with the sinusoidal atom ${\tilde{\phi }}_{k}$ is 2π*k*/*N*. It can be regarded as a representation matrix of mapping: ℂ^*N*^ ↦ ℂ^*K*^. Different from ϕ_*k*_, the definition of ${\tilde{\phi }}_{k}$ is given as

(9) $$\begin{array}{l} \phi_{k}={\left(e^{j\cdot \frac{2\pi }{K}\cdot k\cdot 0},\;e^{j\cdot \frac{2\pi }{K}\cdot k\cdot 1},\;e^{j\cdot \frac{2\pi }{N}\cdot k\cdot 2},\;\ldots ,\;e^{j\cdot \frac{2\pi }{K}\cdot k\cdot \left(N-1\right)}\right)}^{T}. \end{array}$$

The vectors *x*, ϕ_*k*_, ${\tilde{\phi }}_{k}$ are of the same dimension. The redundancy factor of the dictionary of *A* can be defined as *Q* = *K*/*N*. The forward transform of *x* with respect to *A* can be written as *A*^*H*^*x*. However, according to the Fourier theory, this process is somewhat time consuming. By using the algorithm of FFT, a fast implementation can be shown as blow. The first step is to enlarge the length of *x* to the dimension of *K* by zero-padding,

(10) $$\begin{array}{l} x\overset{Zero\;Padding}{\underset{\;}{\to }}\left[\begin{array}{c} x\\ 0_{1\times \left(N-k\right)} \end{array}\right]. \end{array}$$

The second step is to perform FFT on the augmented series. However, it can be seen that this forward transform is generally a dense representation. Therefore, a sparse coding algorithm is required.

### Sparse Fourier Spectrum Analysis via SALSA

In optimization theory, a constraint optimization problem can be summarized as

(11) $$\begin{array}{l} \underset{z}{\arg\min}E\left(z\right)\text{ such that }Cz-b=0, \end{array}$$

where *E*(·) is the cost function. The associated augmented Lagrangian is defined as

(12) $$\begin{array}{l} L_{A}\left(z,\lambda ,\mu \right)=E\left(z\right)\;+\;\lambda^{T}\left(cz-d\right)+\mu \|cz-b{\|}_{2}^{2}. \end{array}$$

where the vector-valued variable λ is the Lagrange multiplier. Based on these methodologies, we can find numerical solutions for sparse representation problems.

Sparse representation (SR) is a novel idea of signal expansion by using redundant dictionaries, which can be expressed as

(13) $$\begin{array}{l} y=Ax. \end{array}$$

In the above equality, the matrix *A* represents a dictionary of the dimension *N* × *K*, in which *K* ≫ *N*. The variable *x* is the linear combination coefficient vector of the dimension *K* × 1. The theory of SR requires that most of the entries in *x* are zero. The solution to the above optimization problem can by numerically implemented via either matching pursuit (MP) or basis pursuit (BP). In this article, we present the idea of sparse Fourier spectrum based on BP algorithms. In engineering applications, in order to handle the noises in the measurement, an improved problem of $P_{1}^{\varepsilon }$ can be formulated as

(14) $$\begin{array}{l} \min\|x{\|}_{1}\text{such that }\|Ax-y{\|}_{2}⩽\varepsilon , \end{array}$$

where ε stands for the admissible error. That is to say, the ℓ_1_ norm is utilized as the measure of the sparsity. In the literature, various techniques have been developed to solve the above $P_{1}^{\varepsilon }$ problem. In this paper, the strategy of split augmented Lagrangian shrinkage algorithm (SALSA) is employed. The strategy of SALSA (20) is celebrated due to its flexibility and fast convergence. By introducing the ideals of variable splitting and augmented Lagrangian (21) into this algorithm, it can address the constraint optimization problem with robust performance. As such, let the observed signal be *y* and the dictionary matrix be *A*, the $P_{1}^{\varepsilon }$ problem to obtain the optimized solution $\hat{x}$ can be written as

(15) $$\begin{array}{l} \underset{x}{\hat{x}=\arg\min}\frac{1}{2}\|y-Ax{\|}_{2}^{2}+\|\lambda ⊙x{\|}_{1}, \end{array}$$

where the vector-valued variable λ is the Lagrange multiplier and the operator ⊙ means element-wise product of two vectors of equal size. The *i*-th element of λ ⊙ *x* is defined as

(16) $$\begin{array}{l} {\left[\lambda ⊙x\right]}_{i}=\lambda_{i}x_{i}. \end{array}$$

Applying the strategy of variable splitting, we can have the following problem.

(17) $$\begin{array}{l} \underset{x,u}{\hat{x}\;=\;\arg\min}\frac{1}{2}\|y-Ax{\|}_{2}^{2}+\|\lambda ⊙u{\|}_{1}\text{ such that }u-x\;=\;0. \end{array}$$

According to the augmented Lagrangian theory, the problem in Equation (17) can be prepared in a matrix form, which is shown as below.

(18) $$\begin{array}{l} z_{1}=x,z_{2}=u,z=\left[\begin{array}{c} z_{1}\\ z_{2} \end{array}\right],C=\left[\begin{array}{cc} I & -I \end{array}\right],b=0. \end{array}$$

and

(19) $$\begin{array}{l} E\left(z\right)=\frac{1}{2}\|y-Az_{1}{\|}_{2}^{2}+\|\lambda ⊙z_{2}{\|}_{1} \end{array}$$

### The Proposed Adaptive Sparse Detector (ASD) for PLI

It can be inferred form the definition of the redundant dictionary *A* that it is a Parserval tight frame because *A*^*H*^*A* = *pI*, where *p* is a constant. According to the theory of SALSA, the following algorithm based on iterations can be employed. The series *y* is the measured EGG signal, which is used as the input of the algorithm. The series *x* is the linear combination coefficient series. The variables λ and μ are necessary parameters required by the algorithm. The procedure of the algorithm is summarized as below.

Step 1. Initialize the parameters and passenger variables: *k* = 1 μ > 0, *d*

Step 2. Repeat the following routine

(20) $$\begin{array}{lll} v & = & soft\left(x+d,\frac{\lambda }{\mu }\right) \end{array}$$

(21) $$\begin{array}{lll} d & = & \frac{1}{\mu +p}A^{H}\left(y-Av\right) \end{array}$$

(22) $$\begin{array}{lll} x & = & d+v \end{array}$$

Step 3. *k* = *k* + 1. If *k* ⩽ *Inter*_*num*, repeat Step 2. Otherwise, the iteration ends.

**Figure 1:**
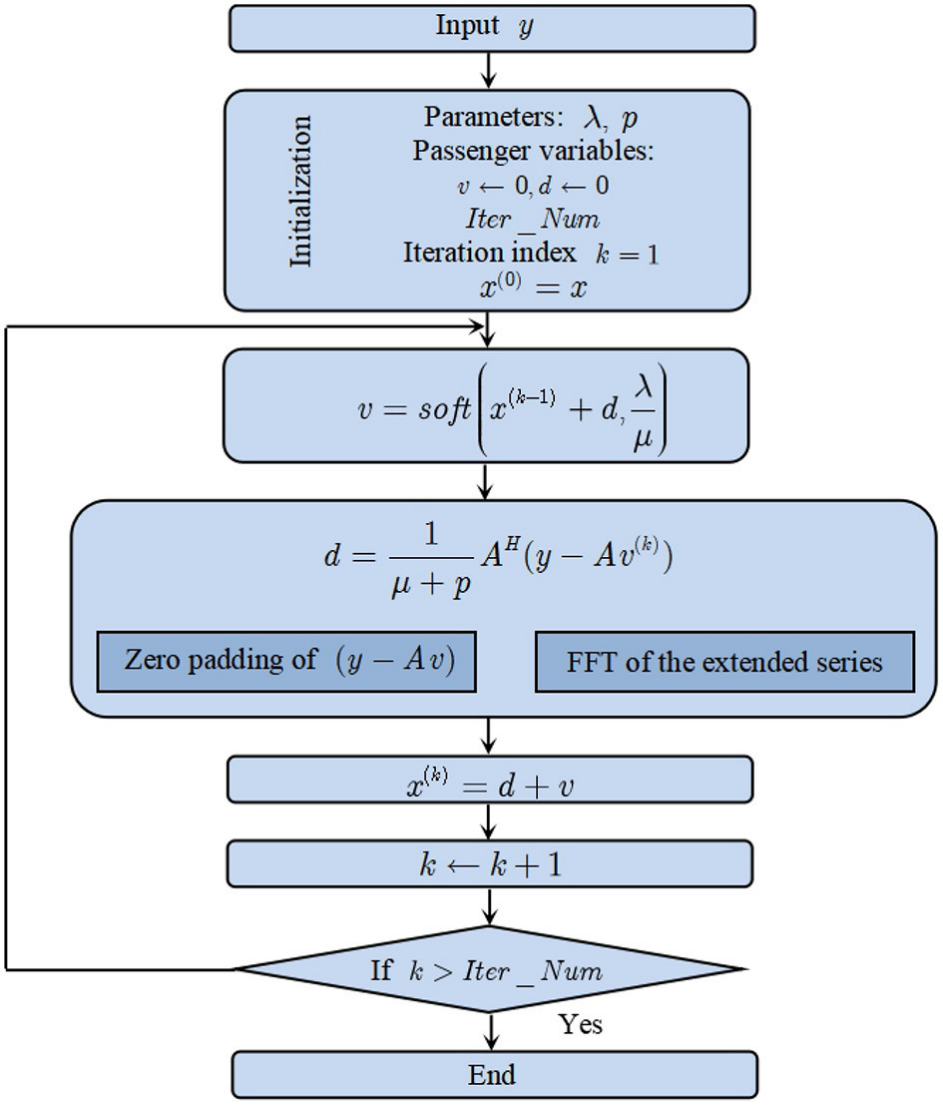
Flow chart of the proposed ASD algorithm.

In the above procedure, the function *soft*(·) indicates the soft threshoding function defined by

(23) $$\begin{array}{l} soft\left(x,T\right)\;=\;\max\left(T./x,0\right), \end{array}$$

in which the symbol ′ − /′ means division by element and *T* is the threshoding value. For the ease of argument, we require that *K*, the number of atoms in the redundant dictionary *A*, to be multiples of *N*. The relationship of *K* and *N* is expressed as *K* = *MN*, in which *M* is a positive integer greater than one. The flowchart of the algorithm is depicted in Figure 1.

## Results

### Numerical Simulation

**Figure 2:**
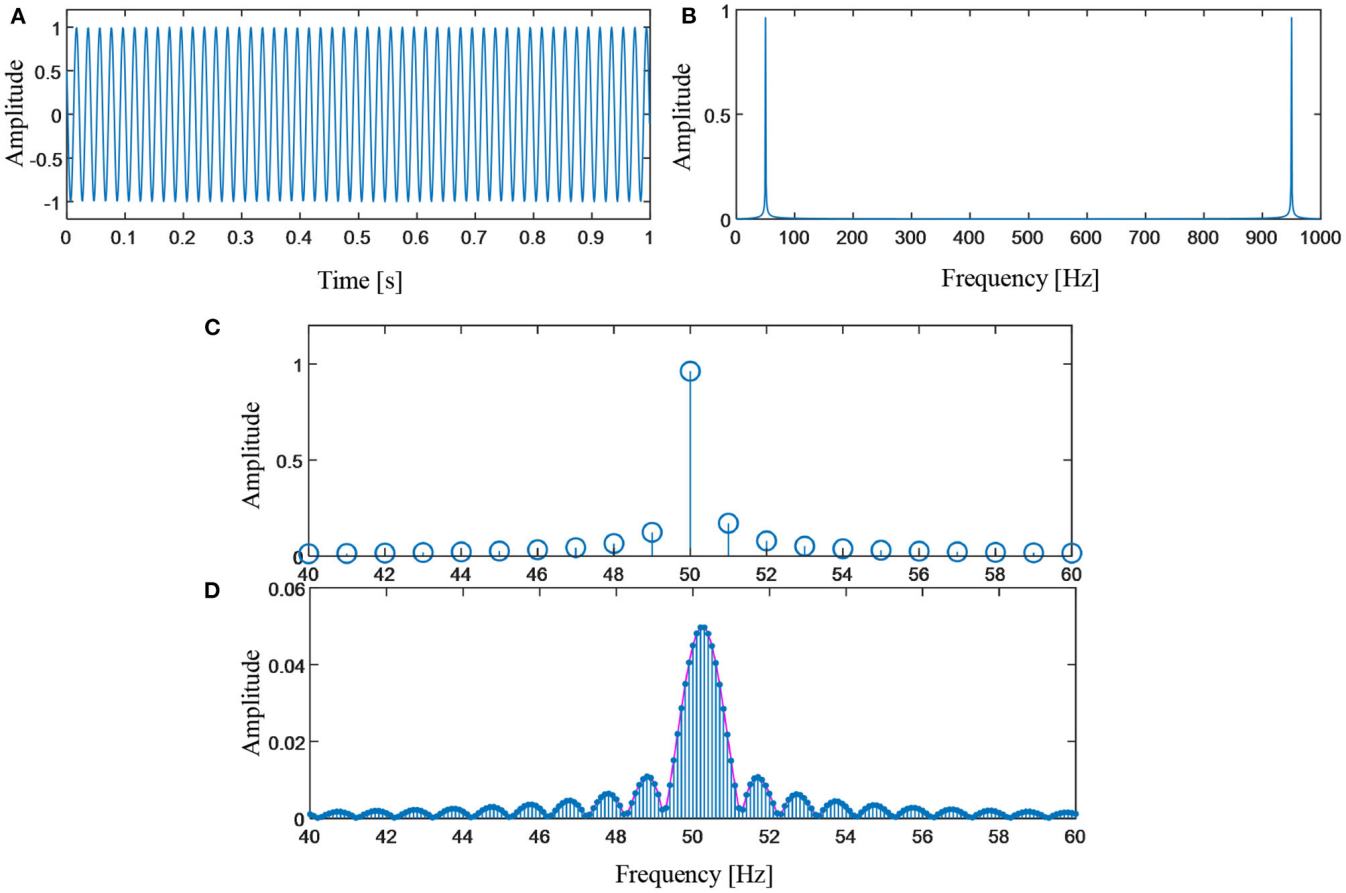
**(A)** Time domain waveform of the simulated signal; **(B)** FFT spectrum of the simulated signal; **(C)** zoom-in plot of the FFT spectrum; and **(D)** linear combination coefficients of the redundant Fourier dictionary.

In this subsection, numerical simulations are utilized to validate the performance of the proposed ASD. Following the definition of PLI in Equation (1), a digital signal is set as

(24) $$\begin{array}{l} p\left(t\right)=\cos\left(2\pi \cdot \left(50+\Delta f\right)\cdot t+\frac{\pi }{3}\right), \end{array}$$

in which the frequency shift Δ*f*=0.15 Hz. The number of samplings and the sampling frequency are set as 1,000 and 1,000 Hz respectively. The time domain waveform of the *p*(*t*) is shown in Figure 2A. In the spectral analysis, the frequency resolution is calculated as.

(25) $$\begin{array}{l} \Delta f=\frac{fs}{N}=1Hz/Spectral\;Line \end{array}$$

Because the sampling of the sinusoidal component does not meet the full period sampling condition, the energy leakage phenomenon occurs in the FFT spectrum. Figure 2B shows the energy of this sinusoidal component spreads across the entire frequency domain with slow decaying rate.

A redundant Fourier dictionary with the definition in Equation (8) is used to represent the signal. The redundancy factor of the dictionary is set as 10. That is to say, ten thousand sinusoidal atoms are employed. A direct transform generates the redundant Fourier spectrum (Figure 2D). Compared with the waveform in Figure 2C, the space between adjacent spectral lines is reduced to one-tenth of that in the FFT spectrum. However, the energy leakage problem remains unchanged, which can be observed from the envelope of the redundant spectrum.

To sparsely represent the signal, the ASD algorithm is performed by using the redundant Fourier basis. The iteration number is set as 100. Let *x*^(*k*)^ be the linear combination coefficients in the *k*-th iteration, an indicator of loss function can be defined as

(26) $$\begin{array}{l} \text{Loss Fun}\left(k\right)={\|\lambda ⊙x^{\left(k\right)}\|}_{1}. \end{array}$$

**Figure 3:**
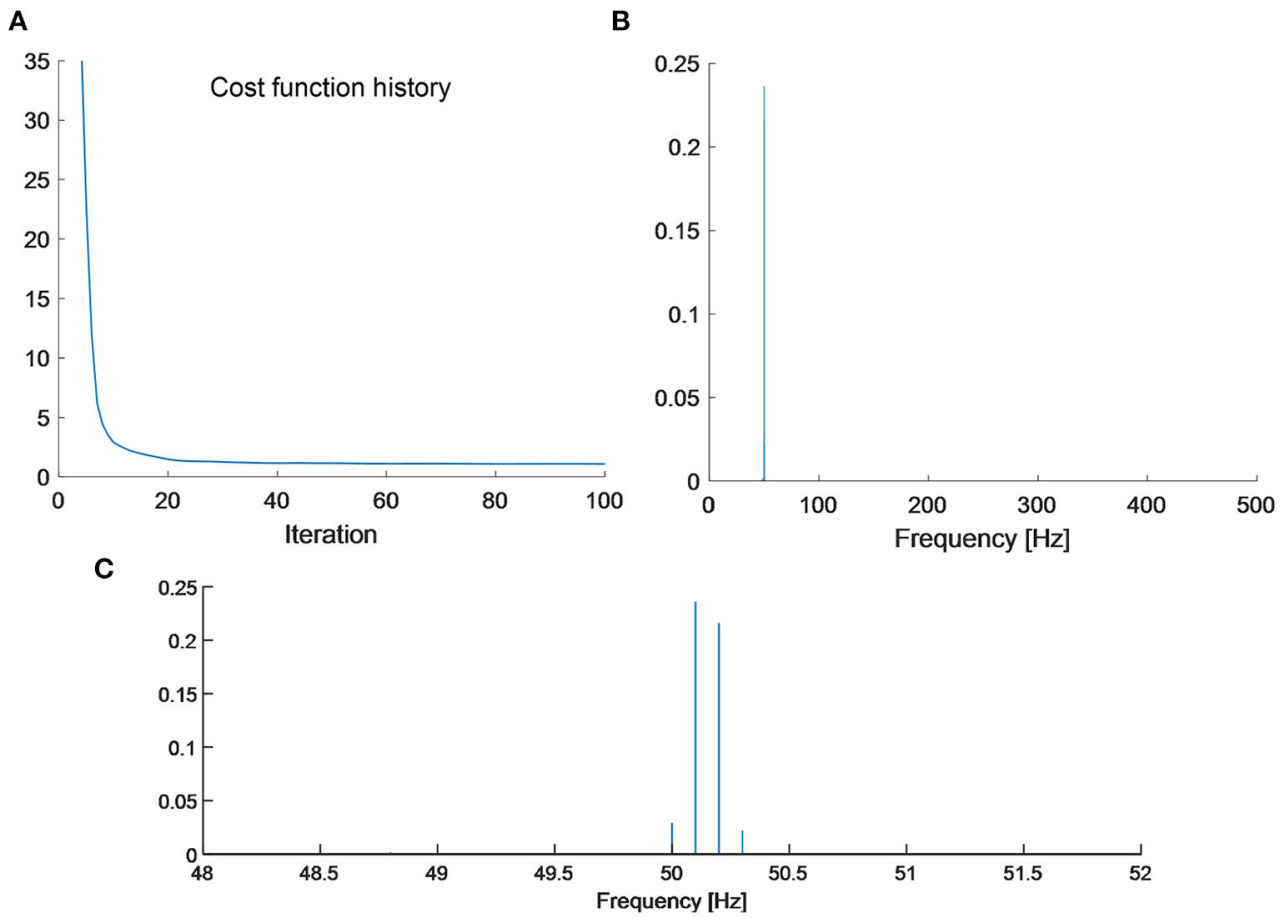
**(A)** The cost function history; **(B)** sparse Fourier spectrum by the proposed ASD methodology; and **(C)** zoom-in plot of sparse Fourier spectrum.

The curve of the cost function is shown in Figure 3A. It can be seen that the cost function converges quickly. About tem times of iterations are enough to guarantee an approximate sparse representation with respect to the dictionary *A*. The final linear combination coefficient vector $\hat{x}$ of the ASD methodology is shown in Figure 3B. It can be observed that only three spectral lines are large in amplitude. The associated frequencies of them are 50, 50.1, 50.2, and 50.3 Hz. The amplitudes of the other spectral lines are very small in value, so they are negligible.

### Processing Results of Actual EEG Measurements

**Figure 4:**
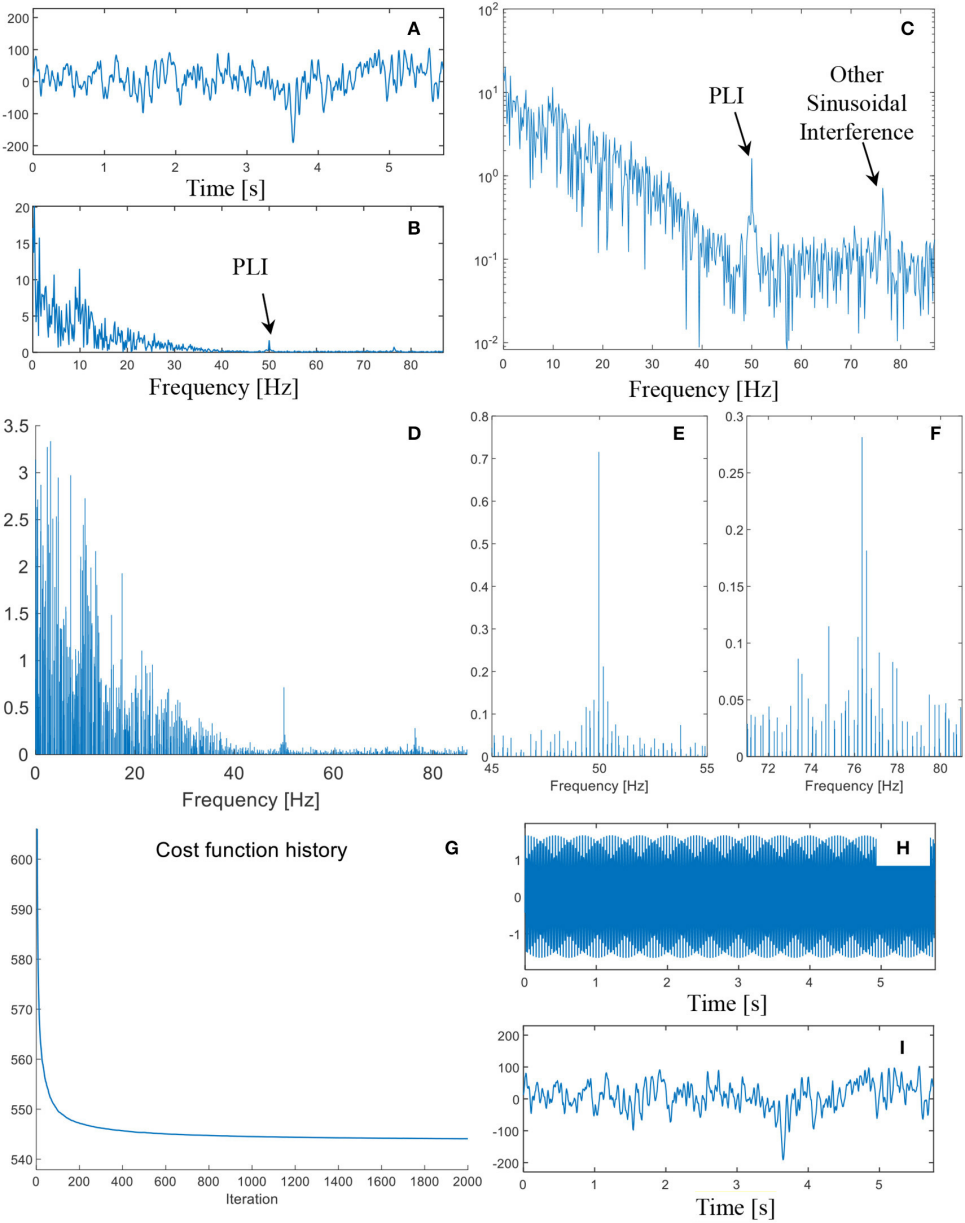
**(A)** The time domain waveform of the EEG measurement; **(B)** FFT spectrum in linear scale; **(C)** FFT spectrum in logarithmic scale; **(D)** sparse FFT spectrum by the proposed method; **(E)** zoom-in plot of the sparse spectrum in the neighbor of 50 Hz; **(F)** zoom-in plot of the sparse spectrum in the neighbor of 76 Hz; **(G)** the cost function history of the iterated algorithm; **(H)** the synthesized compensation signal; and **(I)** the denoised signal.

In this subsection, the analyzed datasets are provided by the Department of Epileptology at University of Bonn (22). The EEGs were recorded at the sampling frequency of 173.61 Hz. According to the Shannon sampling theorem, the spectral band-width of the EEG recordings is 0.5–85 Hz. Digital filters, with the passing band of 0.53–40 Hz, were utilized as pre-processings of the EEGs. Figure 4A illustrates a EEG segment collected from healthy volunteers in an awake state with eyes open. The FFT spectrum of the signal segment is shown in Figure 4B. Due to the band pass filtering step, the spectrum components in the frequency range 40–85 Hz are relatively small. However, by visual inspection, an energy concentration area can be still found near the power line frequency (Figure 4C).

To remove the PLI, the ASD methodology is performed on the EEG segment. In order to guarantee the convergence of the algorithm, two thousand iterations are employed. The convergence of the loss function can be found in Figure 4G. The sparse Fourier spectrum is depicted in Figure 4D. The contents of the signal in the frequency domain are quite complicated. Many non-stationary components can be detected. Due to the sparse representation algorithm, the amplitudes of many spectral lines are almost zero. As such, sinusoidal waves, whose sparse spectrum consists of a very limited number of spectral lines, can be effectively isolated from other non-stationary components.

Two spectral lines, whose frequencies are 49.95 and 49.96 Hz, can be used for retrieve the PLI component. Besides, another strong sinusoidal wave, whose frequency is ~76 Hz, is also found in the spectrum. zoom-in plots of two sinusoidal waves are illustrated in Figures 4E,F. The spectral lines of 49.95 and 49.96 Hz are picked out to reconstruct the PLI component, whose time domain waveform is shown in Figure 4H. By checking the shape, it is confirmed that the sinusoidal wave is perfectly reconstructed. By subtracting the retrieved PLI component from the EEG segment, the denoised signal is shown in Figure 4I. The PLI component is relatively weak in energy, and therefore the denoised signal is very similar to the original EEG segment.

## Discussion

According to the above arguments, it can be concluded that the proposed ASD, which is based on the SALSA, can be utilized as an effective algorithm to retrieve sinusoidal waves.

In spectral analysis of sinusoidal waves of finite digital samples, the ASD can achieve sparse representation. For a sinusoidal wave, the phenomenon of energy leakage occurs if its sampling does not meet the full period sampling condition. In such circumstances, the spectral counterpart of a sinusoidal wave is composed of a main lobe and a few side lobes. The side lobes caused by energy leakage spread across the entire frequency domain. For signal analysis with a segmentation window of the rectangular shape, the side lobes decay very slowly. Reconstruction error by using the main lobe will cause a big error. However, by using a redundant Fourier dictionary containing evenly spaced sinusoidal atoms, it is possible to alleviate this problem. In the numerical simulation, the simple harmonic wave can be sparsely represented by four spectral lines. The amplitudes of two adjacent side lobes are 1.63 × 10^−4^ and 1.39 × 10^−4^respectively. The reconstructed signal can be obtained using the four spectral lines shown in Figure 3C. The relative error between the reconstruction error and the original signal is calculated to be 0.2%. While in the EEG recorded from actual measurements, only two spectral lines are sufficient to allow a reconstruction of PLI with high accuracy. The amplitudes of two adjacent side lobes are 3.88 × 10^−7^ and 3.78 ×10^−7^ respectively. The side lobes also exist in the ASD, but they very small in energy. Therefore, the side lobes can be ignored in the reconstruction process.

As shown in the flow chart (Figure 1), there are many parameters in the SALSA algorithm. The redundancy factor (*Q*) of the employed dictionary and the iteration number can directly affect the sparse Fourier spectrum. In the numerical simulation, there are 4 strong spectral lines when *Q* = 10 and *Iter*_*Num* = 100. Keeping the parameter *Q* unchanged, there can be only 2 strong spectral lines when the iteration number is increased to 1,000. On the other hand, a large value of *Q* is beneficial in prompting the sparsity of the resultant spectrum. However, larger values of *Q* and *Iter*_*Num* requires are more time consuming. To suppress the PLI component as well as other sinusoidal waves, we can set the amplitudes of spectral lines as zero relevant to them and keep other spectral contents unchanged. It is unnecessary to identify their harmonic parameters of amplitude, frequency and phase. However, these parameters can be calculated by using FFT on their reconstruction signals.

Regarding how to determine the number of atoms in the Fourier dictionary, as the number of atoms increases, it will be beneficial to the compression of the main lobe width of the PLI, but it will also affect the efficiency of the whole algorithm, generally ten times the number of atoms of the original is enough.

In addition to the method proposed in this paper, the using of adaptive notch filters for PLI removal has been investigated by the authors (23). The core of the study is ratio-based spectral correction which can extract the spectral information of the PLI components. The difference between the study and ours is that the preceding method does not change the spectral resolution. The information of the PLI components is based on the ratio computation, while the energy leakage of the spectrum faces no mitigation. Our method reduces the phenomenon of overlapping by increasing the spectral resolution. And due to the narrow band property of PLI in the spare representation, they can be more easily isolated from other contents, making its signal information extraction efficient and accurate.

## Data Availability Statement

Publicly available datasets were analyzed in this study. This data can be found here: http://epileptologie-bonn.de/cms/.

## Author Contributions

B-qC and W-fS conceived and designed the classification method and reviewed and edited the manuscript. C-qW and B-xZ performed the experiment. C-qW and B-xZ preprocess and analyzed the data and wrote the manuscript. All authors contributed to the article and approved the submitted version.

## Conflict of Interest

The authors declare that the research was conducted in the absence of any commercial or financial relationships that could be construed as a potential conflict of interest.
